# Location and motion of vaginal pessaries in situ in women with successful and unsuccessful pessary treatment for pelvic organ prolapse

**DOI:** 10.1007/s00192-023-05555-9

**Published:** 2023-04-29

**Authors:** Lars L. Boogaard, Charlotte P. R. Triepels, Luc M. Verhamme, Sander M. J. van Kuijk, Judith J. A. E. Donners, Kirsten B. Kluivers, Thomas J. J. Maal, Mirjam Weemhoff, Kim J. B. Notten

**Affiliations:** 1 Department of Obstetrics and Gynaecology, Radboud University Medical Centre, P.O. Box 9101, 6500 HB Nijmegen, the Netherlands; 2 3D Lab, Radboud University Medical Centre, Nijmegen, The Netherlands; 3 Department of Obstetrics and Gynaecology, Zuyderland Medical Centre, Heerlen, The Netherlands; 4 Department of Clinical Epidemiology and Medical Technology Assessment, Maastricht University Medical Centre+, Maastricht, The Netherlands; 5 Department of Obstetrics and Gynaecology, Maastricht University Medical Centre+, Maastricht, The Netherlands

**Keywords:** Dynamic magnetic resonance imaging, Pelvic organ prolapse, Pessary

## Abstract

**Introduction and hypothesis:**

The objective was to compare the location and motion of pessaries between women with pelvic organ prolapse (POP) with a successful (fitting) and unsuccessful (non-fitting) pessary treatment on dynamic magnetic resonance imaging (dMRI).

**Methods:**

A cross-sectional exploratory study of 15 women who underwent a mid-sagittal dMRI of the pelvic floor at rest, during contraction and during Valsalva with three different types of pessaries. The coordinates of the pessaries cross section, inferior pubic point (IPP) and sacrococcygeal junction (SCJ) were obtained and the location (position, orientation) and the motion (translation and rotation) were calculated. Differences between the groups and between the pessaries within the groups were compared.

**Results:**

Nine women with a fitting pessary and 6 women with a non-fitting pessary were selected. In the non-fitting group, the pessaries were positioned more caudally and rotated more in clockwise direction and descended more, but not significantly, during Valsalva compared with the fitting group. The Falk pessary was positioned more anteriorly in the fitting group and more cranially in the non-fitting group compared with the ring and ring with support pessary.

**Conclusions:**

A non-fitting pessary was positioned more caudally at rest; on Valsalva, it rotated more clockwise and moved more caudally, suggesting that the dynamic characteristics of the pessary might play an important role in its effectiveness. Findings of this study serve as a basis for the development of new pessary designs.

## Introduction

Pelvic organ prolapse (POP) has a major impact on women’s social, physical and psychological well-being [1, 2]. In current daily practice only two effective treatments for women with POP are available: surgery and pessary treatment. Surgery has a 30% risk of recurrent POP [3]. Pessary treatment is a conservative treatment consisting of a relatively simple silicone structure that is inserted into the vagina [4, 5]. In a previous study amongst 680 women with POP, two thirds of women chose pessary treatment as their first-line treatment [6]. In 15–29%, a fitting pessary could not be found [7–10]. A non-fitting pessary can be expelled, is uncomfortable, or may result in temporary side effects [10, 11]. Side effects and improper fit cause 20–50% of the women to discontinue pessary use within 1 year. The majority of these women decide to undergo surgery [12, 13].

Pessaries can be classified as support pessaries and space-filling pessaries [14, 15]. Space-filling pessaries such as the Gellhorn and cube pessary use suction or filling of the vaginal space with a diameter larger than the hiatus [7]. Support pessaries, such as the ring pessary, are designed to elevate the superior vagina by resting in the posterior fornix and using the pubic symphysis as support [7]. A recent study by Hong et al., however, showed that the pubic symphysis plays a limited role in the mechanism of action of the ring pessary based on static MRI data. Instead, owing to the ring pessary resting in the posterior fornix, it is likely that the uterus serves as a lever and thereby plays a role in ring pessary retention [16]. This lever mechanism may help to push the cervix posteriorly, allowing the uterus to rise [7]. Understanding the location and motion in dynamic situations is an essential step toward understanding the mechanism of action of these pessaries and could contribute to improvements in pessary design.

The objective of this study was to compare the location (divided into position and orientation) and motion (divided into translation and rotation) of three different support pessaries in women with a successful and unsuccessful pessary treatment at rest, during contraction and during Valsalva using dynamic magnetic resonance imaging (dMRI). We hypothesised that the pessaries would rotate more and translate more in a caudal direction during Valsalva in patients with an unsuccessful pessary treatment compared with those with a successful pessary treatment and that there will be no difference between the pessaries.

## Materials and methods

A cross-sectional exploratory study among Caucasian women was performed at Maastricht University Medical Centre (MUMC+). This study was approved by the local medical ethics committee in 2015 (Ref. METC152006) and was conducted in accordance with the World Medical Association Declaration of Helsinki on medical research in human subjects.

Women who visited the clinic with the request for a pessary treatment or a clinical check of the pessary treatment were recruited by three gynaecologists. All women gave written informed consent. Inclusion criterium was a symptomatic cystocele and/or descensus uteri a minimum of POP-Q stage 2. Pelvic organ prolapse of a minimum of POP-Q stage 2 was defined as descent of the anterior vaginal wall, the posterior vaginal wall, the vaginal apex (uterine or vaginal vault prolapse) or a combination of these compartments of which the most distal portion of the prolapse was ≤10 mm proximal to or distal to the plane of the hymen [17]. Women were excluded for participation if they had a history of prolapse or incontinence surgery, contra-indications for magnetic resonance imaging (MRI) or an isolated posterior vaginal wall prolapse.

Baseline characteristics collected included age, body mass index (BMI), parity, family history, history of hysterectomy, POP-Q, total vaginal length (TVL) and menopausal state. There were two groups of women based on the success of pessary treatment. One group consisted of 8 women in whom pessary treatment had been successful for at least 6 months. A successful fitting was defined as asymptomatic and continued use without expulsion of the pessary. The other group consisted of 7 women in whom it was impossible to find a fitting pessary. An unsuccessful fitting (further referred to as non-fitting) was defined as an ineffective pessary treatment due to pessary movement or expulsion.

All women underwent a dMRI without rectal or intravenous contrast medium. Participants were asked to empty their bladder prior to the examination. The first dMRI in each woman was performed without a pessary, followed by dMRI with three different support pessaries in a random order: a ring, ring with support and a Falk pessary to evaluate if there are differences in location and motion within support pessary designs. The correct size of each pessary was determined during physical examination. If the pessary was expelled during the dMRI recording, the dMRI was performed again.

A gynaecologist gave the participant instructions during the dMRI to perform three different manoeuvres during the recording: a state of rest, contraction and Valsalva. The same protocol was followed for the three different pessaries in each patient. The dMRI of the pelvic floor was acquired on a Magnetom Skyra 3 T MR scanner (Siemens Healthcare, Erlangen, Germany) with the participant in a supine position. T2-weighted MRI were obtained in the mid-sagittal plane using a spin-echo (SE) sequence with TR/TE 2,000/91 ms; flip angle 150°; slice thickness 7 mm; voxel size 1.37 × 1.37 × 7 mm^3^. Each dynamic scan contained 40 measurements. The total duration of the dMRI was approximately 45 min.

**Fig. 1:**
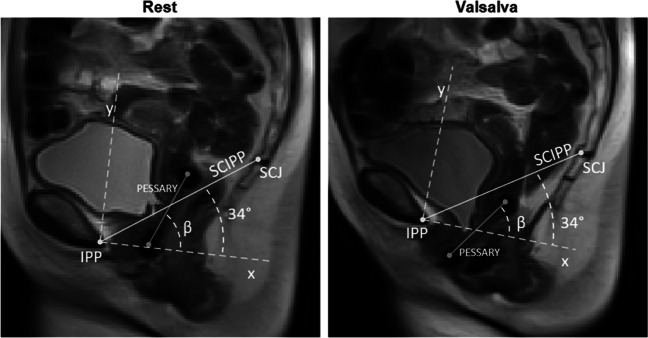
Landmarks on dynamic magnetic resonance imaging showing the pessary angle. The *solid dark grey* represents the pessary and is drawn through the pessary cross-sectional points. The Pelvic Inclination Correction System (*PICS*) line is defined as a line 34° clockwise from the sacro-coccygeal inferior pubic point (*SCIPP*) line (*solid light grey*): a line from the inferior pubic point (*IPP*) to the sacrococcygeal junction (*SCJ*). The origin is located at IPP. The PICS is identical to the x-axis, pointing posteriorly. The y-axis points cranially, perpendicular to the x-axis through the IPP. The definition of the pessary angle (*β*) is the angle between the line through the pessary cross-sectional points (*solid grey*) and the PICS line (*x*)

**Fig. 2:**
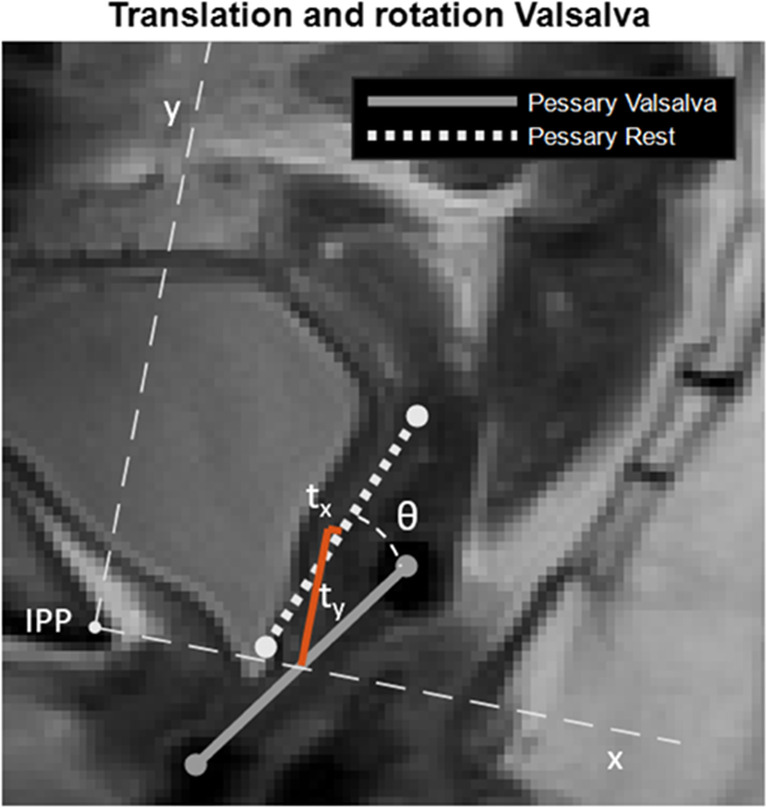
Landmarks on dynamic magnetic resonance imaging showing pessary rotation and translation in x- and y-directions. The *dashed white line* represents the pessary at rest and the *solid grey* at Valsalva (line through the pessary cross-sectional points). The origin is located at the inferior pubic point IPP. The x-axis points posteriorly and the y-axis cranially. The definition of pessary translation in x- and y-directions is shown with t_x_ and t_y_ respectively represented by the *red line*. Pessary rotation from rest to Valsalva is denoted by *θ*

To determine the position, orientation, translation and rotation (Figs. 1, 2 for definition), two examiners (LB, KN) selected the two-dimensional (2D) image corresponding to rest, contraction and Valsalva of each scan. The examiners were blinded to the associated clinical data. The coordinates of the centre of the pessary cross-section (Fig. 1), inferior pubic point (IPP, Fig. 1) and the sacrococcygeal junction (SCJ, Fig. 1) were manually selected by the examiners within the 3D Slicer software (version 4.11, https://www.slicer.org/, Brigham and Women’s Hospital, Boston, MA, USA) [18]. The coordinates of each data point were thereafter exported into MATLAB software (version R2020b, The MathWorks, Natick, MA, USA) for further analysis. The parameters were calculated in the 2D Pelvic Inclination Correction System (PICS) references frame, as described by Betschart et al., which is a local reference frame adjusting for pelvic inclination [19]. The PICS frame has its origin in IPP. The horizontal (x) axis points posteriorly, 34° clockwise with respect to the sacro-coccygeal inferior pubic point (SCIPP) line. The x-axis is thereby, on average, perpendicular to the body axis and referred to as the PICS line. The vertical (y) axis points cranially, perpendicular to the x-axis through the IPP [19].

The orientation of the pessary was defined as the angle between the pessary and the PICS line (Fig. 1). In this study, the orientation was calculated in the sagittal plane only, using the coordinates of the top and bottom cross-section of the pessary. The rotation of the pessary during contraction and Valsalva was defined as the difference in orientation between the pessary at rest and contraction (rotation contraction) and at rest and Valsalva (rotation Valsalva, Fig. 2). The position of the pessary was defined as the coordinates of the pessary inferior cross-section in the PICS reference frame. The translation was defined as the difference in position between the pessary midpoint at rest and contraction (translation contraction) and at rest and Valsalva (translation Valsalva, Fig. 2).

Baseline characteristics are presented as mean ± standard deviation or as number and percentage. The position, orientation, translation and rotation separately for the fitting and non-fitting group, for each pessary, were presented as mean ± standard deviation. Differences between the two groups were tested using (two-sided) independent samples *t* test for continuous variables and Fisher’s exact test for categorical variables. Differences between pessaries within each group were tested using (two-sided) paired samples *t* test. Significance was set at the level of α=0.05. All statistical analyses were performed using IBM SPSS Statistics (version 28.0.0; IBM, Armonk, NY, USA).

## Results

A total of 15 women were included for analysis. One woman was initially included in the non-fitting group because she had masked stress incontinence that became apparent with pessary use. Owing to a correct fitting of the pessary over a 6-month period, it was decided to include the woman in the fitting group for the analysis. Hence, 9 women were included in the fitting group and 6 women in the non-fitting group for the analysis. Owing to technical issues, the dMRI with the ring with support pessary in one woman from the non-fitting group had to be excluded. The baseline characteristics of the study sample are shown in Table 1. Baseline characteristics did not differ between the two groups (all *p*>0.050).

**Table 1:** Baseline characteristics of the total study population, fitting group and non-fitting group

Characteristics	Total study population, (*n*=15)	Fitting group, (*n*=9)	Non-fitting group (*n*=6)	*p* value
Age (years), mean ± SD	69.7±8.5	69.9±9.8	69.3±7.0	0.907
BMI (kg/m^2^), mean ± SD	26.2±3.9	27.8±3.8	23.9±3.2	0.055
Parity, median (range)	2 (1–3)	2 (1–3)	2 (1–2)	0.086
Family history of POP, *n* (%)	6 (40%)	2 (22%)	4 (67%)	0.136
Hysterectomy, *n* (%)	2 (13%)	1 (11%)	1 (17%)	1.000
POP-Q stage 3, *n* (%)	8 (53%)	3 (33%)	5 (83%)	0.119
TVL (cm), mean ± SD	8.4±1.2	8.6±1.5	8.2±0.8	0.253
Postmenopausal, *n* (%)	14 (93%)	8 (88.9%)	6 (100%)	1.000

*BMI* body mass index, *SD* standard deviation, *POP-Q* Pelvic Organ Prolapse Quantification, *TVL* total vaginal length assessed during physical examination

*p* value for the difference between the fitting and the non-fitting group as assessed using the independent samples *t* test or Fisher’s exact test as appropriate

### Pessary position

The pessary inferior cross section was mostly positioned posterior and cranial of the IPP (positive x- and y-position respectively, see Table 2). The ring and Falk pessaries were positioned more caudally at rest in the non-fitting group compared with the fitting group (y-position, *p*=0.022 and 0.025 respectively). Similar results were found for the ring pessary, but without statical significance (*p*=0.054, Table 2). In 3 patients, different pessaries were positioned below the pubic symphysis, all in the non-fitting group.

**Table 2:** Orientation, and x- and y-position by three different support pessaries in the fitting and non-fitting groups

Parameter	Pessary	Fitting (*n*=9)	Non-fitting (*n*=6)	*p* value
Orientation (degrees)	Ring	78.7±12.4	81.0±14.1	0.745
	Ring with support	74.0±11.6^a^	81.3±14.7	0.321
	Falk	73.9±5.1	79.7±18.3	0.483
x-position (mm)	Ring	29.1±6.2	32.3±6.0	0.374
	Ring with support	30.8±4.0^a^	33.5±6.9	0.374
	Falk	26.1±3.8	23.3±10.5	0.480
y-position (mm)	Ring	11.4±4.9	4.7±7.3	0.054
	Ring with support	11.0±4.3^a^	3.9±6.1	0.025
	Falk	7.2±4.9	0.3±5.2	0.022

*p* value for the difference between the fitting and the non-fitting group as assessed by the independent *t* test of the mean ± SD angles, x- and y-positions of each pessary inferior point at rest

^a^Data from one pessary are missing in 1 patient (*n*=8)

The mean x-positions of the pessary inferior cross-section in the two groups were similar. In the fitting group, the mean x-position of the Falk pessary was lower than those of the ring and ring with support pessaries (*p*=0.022 and *p*<0.001 respectively) and the y-position of the Falk pessary was lower compared with the ring pessary (*p*=0.006). In the non-fitting group, the mean y-position of the Falk pessary was lower than that of the ring and ring with support pessaries (*p*=0.013 and *p*=0.019 respectively).

### Pessary orientation

The mean orientation of the pessaries in the two groups were similar, ranging from 64.3 to 106.8° for the fitting group and from 55.0 to 101.8° for the non-fitting group (Table 2). The range of the Falk pessary orientation was higher in the non-fitting group (55.0–101.8°) than in the fitting group (64.4–78.9°). No differences are found between the orientations of the different pessaries in the non-fitting group (all *p*>0.050). In the fitting group, the orientation of the ring was higher than the ring with support pessary (*p*=0.016).

### Pessary translation

In most of the cases, the pessary moved posteriorly and cranially during contraction. In 1 patient with the ring pessary (fitting group), 1 patient with the ring with support pessary (fitting group) and 3 patients with the Falk pessary (2 from the fitting group), the pessary moved anteriorly during contraction. In only 1 case, the ring pessary moved caudally during contraction (fitting group).

Except for 1 patient with a ring pessary (non-fitting group), 1 patient with a ring with support pessary (fitting group) and 2 patients with Falk pessaries (both fitting group), the pessaries moved anteriorly during Valsalva. In all but 1 patient (with a Falk pessary from the fitting group), the pessaries moved caudally during Valsalva.

The ring pessary moved more cranially during contraction, and more caudally during Valsalva in the non-fitting group compared with the fitting group (11.5 vs 4.0 mm, *p*=0.045 and −20.6 vs −12.6 mm, *p*=0.044 respectively). No significant difference was found in the translation in the x-direction during contraction and Valsalva for either pessary between the two groups (Table 3).

**Table 3:** Rotation and x- and y-translation during contraction and Valsalva by three different support pessaries in the fitting and non-fitting groups

		Fitting (*n*=9)	Non-fitting (*n*=6)	*p* value
Rotation contraction (degrees)				
Ring		−0.3±5.9	−6.0±10.2	0.188
Ring with support		−0.1±5.1^a^	−5.3±11.5	0.346
Falk		1.8±8.4	−0.1±14.1	0.746
Rotation Valsalva (degrees)				
Ring		−0.3±5.8	−15.1±21.4	0.066
Ring with support		3.4±8.3^a^	−7.8±12.9	0.070
Falk		−1.8±8.2	−12.7±13.7	0.075
Translation contraction (mm)				
x	Ring	−2.9±6.3	−5.3±6.6	0.478
	Ring support	−5.1±6.2^a^	−7.5±6.5	0.503
	Falk	−6.1±6.2	−5.1±8.7	0.802
y	Ring	4.0±6.1	11.5±7.0	0.045
	Ring support	8.0±3.6^a^	11.7±10.4	0.432
	Falk	6.7±5.0	13.3±7.5	0.062
Translation Valsalva (mm)				
x	Ring	7.3±4.6	5.6±5.5	0.539
	Ring support	5.9±4.7^a^	6.1±3.8	0.933
	Falk	5.4±5.9	11.5±7.2	0.092
y	Ring	−12.6±9.6	−20.6±10.0	0.044
	Ring support	−12.5±3.9^a^	−18.7±9.7	0.124
	Falk	−11.8±5.2	−18.5±10.3	0.183

*p* value for the difference between the fitting and non-fitting groups as assessed by the independent *t* test of the mean ± SD rotation, x- and y-translation of each pessary during contraction and Valsalva

^a^Data from one pessary are missing in 1 patient (*p*=8)

The Falk pessary translated more in the x-direction during Valsalva than the ring and ring with support pessary in the non-fitting group (*p*=0.049 and *p*=0.036 respectively). In the non-fitting group, the mean x-translation of the ring pessary during contraction was less than that of the ring with support pessary and the y-translation of the ring with support was less than that of the Falk pessary (*p*=0.028 and *p*=0.025 respectively). In the fitting group, no differences in translation of the different pessaries are found (all *p*>0.050).

### Pessary rotation

There was no significant difference in the mean rotation of the pessaries from rest to contraction between the two groups. All three pessaries rotated more clockwise during Valsalva in the non-fitting group, but not statistically significantly (*p*=0.066, 0.070 and 0.075 for the ring, ring with support and Falk pessary respectively, Table 3). No differences are found between the rotations of the different pessaries within either the fitting or the non-fitting group (all *p*>0.050).

## Discussion

### Main findings

In this study, we found that the pessaries are positioned more caudally at rest in the non-fitting group. During Valsalva, the pessaries rotated more clockwise and moved caudally in the non-fitting group, but not statistically significantly. Differences between the Falk pessary and the ring and ring with support pessaries are found in the x-position within the fitting group, and in the y-position within the non-fitting group.

### Strengths and limitations

To our knowledge this study is the first attempt at comparing the location and motion of pessaries in women with a fitting and non-fitting pessary using dMRI. All known studies focused primarily on baseline predictors for a non-fitting pessary (i.e. clinical, demographic and anatomical parameters).

Results of this study are intuitive and accurate, as the measurements are corrected for pelvic movement and thereby standardised between patients owing to the usage of the PICS reference frame. By using this standardised frame of reference, the results of this study are suitable for future comparisons.

A limitation of this study is the small sample size. It was set up as an exploratory study with the aim of increasing our understanding of the pelvic floor and pessary treatment. By increasing understanding, it is possible to generate new hypotheses that are worth studying more extensively. Hence, interpretation of effect sizes must prevail over significance of statistical tests, for which power is by design too low. Another limitation of the study is the difficulty in standardising the level of contraction and Valsalva on dMRI, despite a strict protocol being used. The landmarks are detected manually by the examiners, which may be standardised in future studies. Furthermore, the level of contraction and Valsalva performed by the participant may differ because of the difficulty of these manoeuvres for some women.

### Interpretation

The results presented in this study imply that there is less support or stability of the pessaries, especially in the caudal direction in the case of a non-fitting pessary. The large variation in location and motion are in line with the complex biomechanics of the pelvic floor, the variety in morphology and/or aetiology of POP, and the variety in effectiveness of pessary treatment.

The antero-caudal motion found during contraction and posterior-cranial motion during Valsalva are in accordance with anatomical knowledge and biomechanical studies of the pelvic floor. In vivo imaging measurements show that the mean line-of-action (i.e. muscle fibre direction) of the levator ani muscle are mainly in the antero-posterior direction, closing the levator hiatus [20, 21]. During Valsalva, the intra-abdominal pressure increases, resulting in a force acting from the abdominal cavity on the uterus and pessary pointing caudally. The second action of the levator ani muscle is to generate a lifting force, against the action of gravity and counteracting increased pressure from the abdominal cavity [20]. The increased caudal motion of the pessaries during Valsalva found in the non-fitting group could be the result of a decreased lifting force of the levator ani muscles (i.e. in a cranio-caudal direction).

The orientation of the ring pessary found in this study differs slightly compared with those found in Hong et al. [16], who performed an MRI study in fitting pessaries. In their study a mean (sagittal) angle of 57.0° at rest was found based on 21 MRI of patients with a fitting pessary, compared with 78.7° in this study. The position of the inferior cross section of the ring pessary was comparable in the fitting group of this study and in Hong et al. The findings in this study are in agreement with the statement made by Hong et al. that support pessaries (i.e. ring pessaries) do not support against the pubic bone, as the inferior cross-section of the pessaries was not near the pubic bone [16]. This statement is further confirmed by the position found of the pessary inferior cross section, which suggests that there might be no support of the pubic bone.

Findings of this study can be used to improve our understanding of POP and the mechanism of action of pessaries. Results of dynamic imaging studies could be implemented in biomechanical studies to investigate the relation between the motion of the pessary and internal (muscle) forces.

Our findings of pessary movement during Valsalva suggest that using the uterus as lever might be inadequate for elevating pelvic organs in a large part of the patient population, highlighting the need for new pessary designs. New pessary designs should focus on providing sufficient support by limiting the pessary mobility (i.e. rotation) in order to better support the pelvic organs.

Additionally, this study generates new hypotheses regarding predictive parameters for a non-fitting pessary, such as the maximal caudal position and movement allowed by a pessary. To investigate parameters that predict a non-fitting pessary, the dynamic characteristics can be investigated in a larger sample size with less generic groups, e.g. grouping for different types of prolapse (i.e. cystocele, rectocele), size of genital hiatus and/or POP-Q stage. It would be less expensive and clinically more feasible to assess the dynamic characteristics in a consultation room using ultrasound or physical examination rather than dMRI.

This study is a next step towards understanding the mechanism of action of support pessaries. It serves as a basis for and provides insight into the development of new pessary designs with improved stability. The research group is currently undertaking further research into a new pessary design. Further research on the interaction between support pessaries and internal (muscle) forces is recommended, using 3D dMRI with a special interest in muscle visualisation, or the integration of 3D dMRI data of the pessary into a biomechanical pelvic floor model.

## Conclusion

Our study is the first to describe and compare the dynamic characteristics of fitting and non-fitting pessaries. We found that a non-fitting pessary was positioned more caudally at rest and rotated more clockwise and moved more caudally during Valsalva. These results suggest that the dynamic characteristics of the pessary might play a role in its effectiveness, and serve as a basis for the development of new pessary designs.

## Data Availability

The data that support the findings of this study are available from the corresponding author, LB, upon reasonable request.
